# Effect of small dose esketamine on perioperative neurocognitive disorder and postoperative depressive symptoms in elderly patients undergoing major elective noncardiac surgery for malignant tumors: A randomized clinical trial

**DOI:** 10.1097/MD.0000000000040028

**Published:** 2024-10-18

**Authors:** Cuifang Huang, Ruimin Yang, Xianlong Xie, Huijun Dai, Linghui Pan

**Affiliations:** a Department of Anesthesiology, Guangxi Medical University Cancer Hospital, Nanning, Guangxi, China; b Department of Anesthesiology, The First Affiliated Hospital of Guangxi Medical University, Nanning, Guangxi, China.

**Keywords:** elderly patients, esketamine, perioperative neurocognitive disorder (PND), postoperative delirium (POD), postoperative cognitive dysfunction (POCD), postoperative depressive symptoms

## Abstract

**Background:**

Perioperative neurocognitive disorder and postoperative depressive symptoms are significant complications after surgery. Studies have indicated that esketamine possesses neuroprotective and antidepressant qualities.

**Methods:**

This trial included 209 patients aged 60 to 86 years undergoing tumor resection who received esketamine (Group E) or not (Group C) during and after surgery. In group E, patients were given an intravenous dose of 0.5 mg/kg of esketamine 10 minutes after induction of general anaesthesia. In addition, esketamine (2 mg/kg) in combination with sufentanil was used for PCIA during 48 hours postoperatively. On the other hand, saline was used as a substitute for esketamine in group C. Cognitive function was evaluated using neuropsychological tests and telephone interview for cognitive status-modified, and symptoms of depression were assessed using Hamilton Depression Rating Scale 17.

**Results:**

Compared to Group C, patients in Group E exhibited lower rates of depressive symptoms at 3, 7, and 90 days post-surgery (53.9% vs 67.7%, 26.3% vs 47.9%, and 13.3% vs 28.4%). Group E also showed decreased time for Trail Making Test on days 7 and 90. However, there were no significant differences in the incidence of delirium 1 to 5 days post-surgery or cognitive impairment 90 days post-surgery between the 2 groups (12.1% vs 10.9% and 8.4% vs 9.7%).

**Conclusions:**

Intraoperative low-dose esketamine and postoperative low-dose esketamine combined with sufentanil for patient-controlled intravenous analgesia has been shown to improve postoperative analgesia, alleviate postoperative depressive symptoms, and aid in the recovery of social executive ability. However, this approach did not reduce the incidence of postoperative delirium or postoperative cognitive dysfunction.

## 1. Introduction

To date, cancer is the second leading cause of death worldwide after cardiovascular diseases, surgical resection remains one of mainstay treatments for most cancers. Perioperative neurocognitive disorder (PND) is a severe complication following surgery and anesthesia in elderly patients. It is usually manifested by disturbances of the cognitive ability, orientation ability, thinking, emotion and sleep, and often accompanied by dysfunction of personality, social ability and communication skills, and other social activities of the ability. PND includes cognitive decline diagnosed before the operation; postoperative delirium (POD), delayed neurocognitive recovery (up to 30 days after the procedure) and postoperative neurocognitive disorder (up to 12 months, POCD).^[1]^ Due to the increase in the aging population, half of the elderly globally have received a surgical procedure. A quarter of those who undergo major surgery will experience noticeable cognitive decline, and half of all patients will suffer from permanent cognitive impairment.^[2]^ PND results in more extended hospitalization, increased disability, social burden, and mortality. However, effective pharmacotherapies to prevent or reduce PND are not currently available.

Depressive symptoms are the most common comorbid disease among elderly cancer patients. It is characterized by symptoms of sadness, depressed mood, and loss of interest. Depressive symptoms is one of the most prevalent mental disorders in the world.^[3]^ Patients with cancer and depressive symptoms are associated with a poor prognosis and a high mortality rate. It has been reported that depressive symptoms in patients treated for cancer are more than 5 times that observed in noncancer patients.^[4]^ Depression increases the sensitivity of the patient’s body to pain,^[5]^ decreases the patient’s autoimmunity,^[6]^ and leads to a prolonged postoperative recovery time,^[7]^ Besides, depressive symptoms accelerated cognitive decline in elderly people.^[8–10]^

Esketamine (S-ketamine) is dextroketamine, which is isolated and purified from ketamine. It has a higher affinity with N-methyl-D-aspartate receptor and μ-opioid receptors than ketamine, as well as a stronger analgesic effect.^[11]^ In terms of adverse effects, esketamine is similar to ketamine but may be higher than aketamine (e.g. dissociative effects).^[12]^ Esketamine is known to be a psychoactive drug with hallucinogenic properties that could theoretically contribute to the occurrence and development of PND, but previous research had reported potential neuroprotective effects of esketamine.^[13,14]^ Recently, The US Food and Drug Administration (FDA) recently approved S-ketamine nasal spray as a novel treatment for drug-resistant depression and adults with major depression associated with acute suicidal ideation or behavior.^[11]^ Prolonged and repeated use of esketamine nasal spray may lead to a higher occurrence of adverse events. Along with common side effects like dizziness, dissociation, nausea, drowsiness, and headache, there may also be concerns regarding addiction and suicidal tendencies.^[15–17]^ This emphasizes the importance of closely monitoring the risks and negative effects linked to esketamine in a clinical environment. In addition, esketamine has been shown to relieve pain,^[18,19]^ save on opioid use,^[20,21]^ and improve sleep.^[22]^

Based on a synthesis of existing evidence, our findings suggest esketamine as a promising adjunctive non-opioid therapy to reduce postoperative depressive symptoms, postoperative pain, and the consumption of opioids, which ultimately reduces the incidence of PND. However, this drug has not been tested clinically to prove that these effects can still be observed perioperatively in elderly cancer patients undergoing major noncardiac surgery. Therefore, this study aimed to evaluate the effect of esketamine on perioperative neurocognitive disorder and perioperative depressive symptoms in elderly patients with malignant tumors undergoing major elective noncardiac surgery.

## 2. Material and methods

This clinical study was carried out at Guangxi Medical University Cancer Hospital from August 1, 2022 to August 30, 2023 and approved by the medical ethics committee of Guangxi Medical University Cancer Hospital [number: KY2021288] and registered with the Chinese Clinical Trial Registry (NO. ChiCTR2300069249). Each study subject provided written informed consent before the first visit.

### 2.1. Patients recruitment

The inclusion criteria included patients ≥60 years of age, years of education is more than 6, American Society of Anesthesiologists physical status I–III, undergoing surgery involving the orthopedic, urologic and major abdominal surgeries who were diagnosed as cancer in pathological report and with an anticipated length of hospital stay of at least 5 days after surgery, and willing to complete the neuropsychological tests. Exclusion criteria were preexisted neurological disorders, muscular or endocrine diseases or psychiatric diseases (Alzheimer disease, Parkinson disease, or dementia), serious medical diseases (heart failure, respiratory failure, pulmonary hypertension, liver and kidney dysfunctions, brain metastasis of cancer), unwell to accept analgesia or unable to understand numerical rating scale (NRS), or operate patient-controlled analgesia, severe language, visual or auditory deficiency, planned postoperative intubation, telephone interview for cognitive status-modified (TICSm) score <27 and known allergies to any of the other drug used in the protocol.

### 2.2. Randomization and blinding

A computer-generated random number sequence was used to randomly assign patients to receive either an perioperative esketamine infusion or equivalent saline. The patients were undergoing resection of cancer tumors of various types. The assignment ratio was 1:1. The esketamine infusion dosage was 0.5 mg/kg intraoperatively and 2mg/kg postoperatively combined with sufentanil for patient-controlled intravenous analgesia (PCIA). Eketamine 50 mg was diluted to a final volume of 5 ml with saline, and an equivalent volume of saline was also prepared for the control group. Group assignments were placed in sealed envelopes to conceal allocation and given sequentially to nurses not involved in the study. The anesthesiologists involved in patient management and postoperative follow-up were unaware of the groupings. Group assignments were not announced until after the patient was discharged.

### 2.3. Interventions and perioperative management

Prior to surgery, intestinal preparation and abstinence from drinking and fasting were conducted without any preoperative medication. Various vital sign measurements, including electrocardiograph, heart rate, respiratory rate, blood pressure, arterial blood pressure, central venous pressure, oxygen saturation, and bispectral index were closely monitored upon admission. General anesthesia was induced through intravenous administration of midazolam 0.02 to 0.05 mg/kg, propofol 1.5 to 2 mg/kg, sufentanil 0.4 μg/kg, and rocuronium 0.6 mg/kg, and maintained through propofol 4 to 12 mg/kg^-1^·h^-1^, remifentanil 0.1 to 0.3 μg/kg^−1^·min^−1^, with intermittent intravenous injections of rocuronium bromide to sustain muscle relaxation. In Group E, patients received esketamine at a dose of 0.5 mg/kg (diluted to 10 mg/mL) intravenously 10 minutes after the induction of general anesthesia. Additionally, esketamine (2 mg/kg), sufentanil (2 μg/kg), flurbiprofen (2 mg/kg), and tolansetron (0.2 mg/kg) were administered in 100 mL of saline for PCIA over the 48-hour postoperative period. On the other hand, in Group C, saline was used as a substitute for esketamine. Specifically, a volume of saline equivalent to 0.05 mL/kg was injected 10 minutes after the induction of general anesthesia. Sufentanil (2μg/kg), flurbiprofen sodium (2 mg/kg), and tolansetron (0.2 mg/kg) were then administered in 100 mL of saline for PCIA over the 48-hour postoperative period. An IV analgesia pump was used for patient-controlled administration, with a background infusion of 2 mL/h for 48 hours, a bolus of 2 mL, and a lockout time of 10 minutes. If the patient’s blood pressure was below 30% of the basal blood pressure, 5 to 10 mg ephedrine hydrochloride was injected. If the patients had bradycardia during the operation and their heart rate was below 50 beats/min, atropine 0.25 to 0.5 mg was administered. When the NRS score on movement was ≥3, additional diazoxide (5 mg) was added.

### 2.4. Neuropsychological tests

#### 2.4.1. Delirium battery

POD was evaluated using the confusion assessment method. POD was assessed twice daily for 7 days postoperatively, once in the morning (7:00–8:00) and once in the afternoon (19:00–20:00). The interval between the 2 assessments should not be <6 hours. The content includes 4 aspects: (1) acute onset and fluctuations in the course of the illness, (2) inattention, (3) incoherent thinking, and (4) alterations in consciousness. Delirium can be diagnosed by having both (1) and (2) and either (3) or (4).^[23]^

#### 2.4.2. Cognitive battery

The neurocognitive evaluation included 4 cognitive domains: memory, attention, language, and executive function. The memory domain includes Digit Span Task backward, the attention domain includes Stroop Color Word Test Part 3 and Symbol Digit Modalities Test, Languages include language fluency Test speech part categories, Areas of executive function include Trail Making Test part B. Patients were assessed 1 day before surgery (baseline) and 7 days after surgery (If discharged in fewer than 7 days, conduct testing on the day of discharge). Cognitive change is assumed if the difference between 2 or more neuropsychological tests before and after surgery is greater than 1 standard deviation.^[24]^ Hospitalized patients can only receive early cognitive function assessments. At 7 and 90 days post-surgery, a TICSm was conducted, which included 12 questionnaires, and the overall cognitive function was assessed by verbal communication. Ratings range from 0 to 50,^[25]^ with higher scores indicating better functionality, a total score ≤27 for dementia, and <32 for mild cognitive impairment (MCI). It mainly includes spatial and temporal orientation, mental control, memory, general information, language, and calculation ability.^[26]^ The same trained researcher performed all neurocognitive assessments. A physician in the neurology department supervised the quality of the assessments.

### 2.5. Depression

Hamilton Depression Scale 17 (HAMD-17) is a 17-item tool used to determine the severity of depressive symptoms. A higher score indicates a higher severity of depression. HAMD-17 score ≥ 7 classified as depressive symptoms.^[27]^

### 2.6. Outcome measurement

The primary endpoint was the incidence of POD and POCD in both groups. Secondary endpoints included: (1) incidence of MCI on day 90 after surgery; (2) incidence of postoperative depressive symptoms; (3) postoperative patient recovery quality.

### 2.7. Statistical analysis

#### 2.7.1. Sample size

The sample size was calculated based on a clinical trial showing a POCD incidence of approximately 40% 7 days after major noncardiac surgery.^[28]^ The incidence of POCD was expected to decrease from 40% in Group C to 20% in Group E. For an α-error of 0.05 (2-sided) and a power of 90%, at least 78 patients were assigned to each group. Considering that approximately 25% of patients would be lost to follow-up at 7 days post-operation, 105 patients were recruited in each group.

### 2.8. Data analysis

The SPSS 20.0 software package (Armonk, NY) was used for the statistical data analysis. Normally distributed measures were expressed as mean ± standard deviation (x ± s) and compared between groups using independent samples *t* test or one-way ANOVA. Skewed measures were expressed as median (M) and interquartile range and compared between groups using Mann–Whitney *U* test. Count data were expressed as rates (%), and comparisons between groups were made using the 2 test (Pearson 2 test when n ≥ 40 and T ≥ 5; continuity-corrected 2 test when n ≥ 40 or 1 < *R* < 5; Fisher exact when n < 40 or F ≤ 1 probability test). A *P* < .05 indicate a statistically significant difference.

## 3. Results

**Figure 1.**
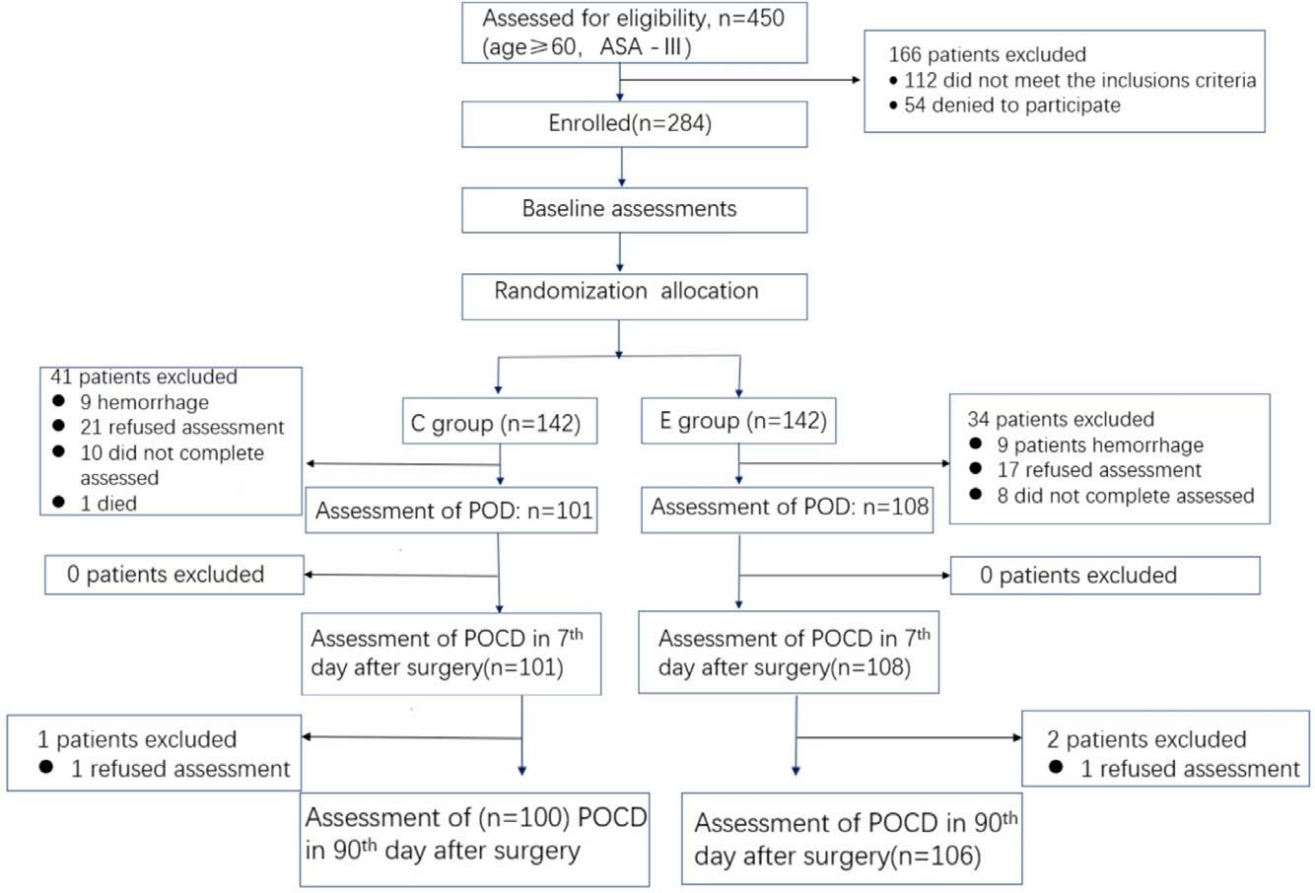
Flowchart of the study. POD, postoperative delirium; POCD, postoperative cognitive dysfunction.

A total of 450 patients were screened, of which 166 met the exclusion criteria, and 284 signed a consent form to participate in this clinical trial (142 in Group E and 142 in the control group). On postoperative day 1 to 5, 41 patients in Group C withdrew from the study, including 7 with hemorrhage, 10 who could not complete the test independently, 23 who refused cognitive function assessment, and one who died of multiple organ failure syndrome on postoperative day 5. Thirty-four patients in Group E withdrew from the study, including 9 with hemorrhage, 8 who could not complete the test independently, 17 who refused cognitive function assessment, and 2 who refused neuropsychological testing on postoperative day 90. On postoperative day 7, no patients in either group withdrew from the trial, and on postoperative day 90, 1 patient in Group C refused to undergo neuropsychological testing, and 2 patients in Group E refused (Fig. 1). There were no statistical differences between the groups. Demographic data and preoperative clinical characteristics are shown in Table 1.

**Table 1:** Demographic and baseline data in patients.

Variable	E group (N = 108)	Control group (N = 101)	*P*-value
Age, median (IQR), year	63.0 (62.0, 66.0)	63.0 (62.0, 66.0)	.93
Gender, n (%)			
Male	46 (42.6)	49 (48.5)	.39
Female	62 (57.4)	52 (51.5)	
BMI, median (IQR), kg/m^2^	22.8 (20.8, 25.0)	22.5 (20.5, 25.5)	.69
ASA physical status, n (%)			.62
Class I	12 (5.6)	8 (3.9)	
Class II	174 (81.7)	166 (81.4)	
Class III	27 (12.7)	30 (14.7)	
TNM stage, n (%)			.47
I	71 (65.7)	61 (60.4)	
II	37 (34.3)	40 (39.6)	
Chemotherapy	23 (21.3)	24 (22.7)	.67
Educational level, median (IQR), year	8 (6,11)	8 (6,11)	.66
Chronic smoking, n (%)	31 (28.7)	23 (22.8)	.47
Alcoholism, n (%)	25 (23.1)	19 (21.3)	.49
Preoperative depressive symptoms, n (%)	32 (29.6)	37 (36.6)	.28
Comorbidity, n (%)			
Hypertension	29 (26.9)	28 (27.7)	.51
Diabetes	10 (9.3)	10 (9.9)	.53
Arrhythmia	16 (16.8)	20 (19.8)	.36
Anemia	6 (5.6)	9 (8.9)	.43
The history of surgery	39 (36.1)	44 (43.6)	.32
Fatigue, n (%)	21 (19.4)	15 (14.9)	.24

*Notes*: The results are presented as the number (%) or median (interquartile range).

ASA = American Society of Anesthesiologists, BMI = Body Mass Index.

### 3.1. Assessment of postoperative delirium

**Figure 2.**
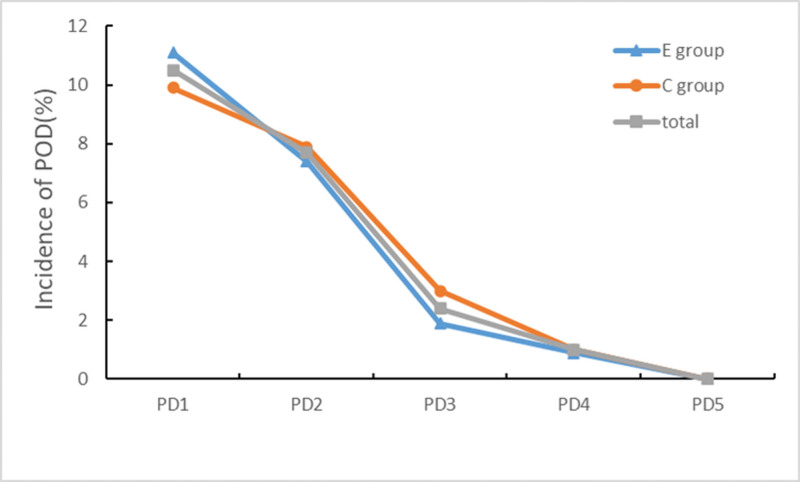
Incidence of postoperative delirium 1 to 5 days after surgery in 2 groups. PD = postoperative X day.

The incidence of delirium during postoperative 5 days is presented in Figure 2. Among these patients, a total of 24 (11.5%) patients developed POD, including 13 (12.1%) in the E group and 11 (10.9%) in the control group, and there was no statistical difference in the incidence of POD between the 2 groups (OR = 0.80; 95% CI, 0.33–1.92; *P* = .62).

### 3.2. Evaluation of cognitive function

On the day before surgery, both groups exhibited similar TICSm scores and individual test scores on neuropsychological assessments. By the 7th day after surgery, TICSm scores had slightly decreased in both groups, but by the 90th day post-surgery, TICSm scores had returned to baseline levels with no significant difference observed between the groups. The MCI and POCD incidences at 7 and 90 days after surgery were not statistically significant in the 2 groups. In neuropsychological tests, the E group took less time than the control group to Trail Making Test (*P* < .05) see Table 2.

**Table 2:** Comparison neuropsychological test, TICSm and incidence of MCI and POCD.

	E group	C group
Digit span test (backward)		
Baseline	2.0 (2.0, 3.0)	2.0 (2.0, 3.0)
PD7	2.0 (2.0, 3.0)	2.0 (2.0, 3.0)
PD90	2.0 (2.0, 3.0)	2.0 (2.0, 3.0)
Symbol Digit Modalities Test		
Baseline	24.0 (22.0, 26.0)	25.0 (22.0, 25.0)
PD7	23.0 (21.7, 25.0)	22.0 (20.0, 24.0)*
PD90	23.0 (21.0, 25.0)	22.0 (20.0, 24.0)*
Stroop Color Word Test (Part 3)		
Baseline	79.0 (67.7100.1)	75.0 (65.0, 90.0)
PD7	85.0 (72.0, 105.2)*	79.0 (70.1, 96.0)*
PD90	85.5 (68.7100.0)*	79.0 (68.0, 92.0)*
Verbal Fluency Test (speech)		
Baseline	4.0 (3.0, 4.0)	3.0 (2.0, 4.0)
PD7	3.0 (2.0, 4.0)	2.0 (2.0, 3.0)*
PD90	3.0 (2.0, 4.0)	3.0 (2.0, 4.0)
Trail Making Test (Part B)		
Baseline	62.0 (54.0, 69.0)	63.0 (56.0, 66.0)
PD7	65.0 (57.5, 69.2)*	68.0 (62.0, 69.0)*†
PD90	63.5 (56.0, 69.0)	65.0 (59.0, 69.0)*†
TICSm		
Baseline	33.0 (33.0, 35.0)	33.0 (33.0, 35.0)
PD7	33.0 (32.0, 34.0)	33.0 (30.0, 34.0)
PD90	33.0 (33.0, 35.0)	34.0 (33.0, 35.0)
MCI, n (%)		
PD7	36 (33.3)	39 (38.6)
PD90	16 (14.8)	22 (21.8)
POCD, n (%)		
PD7	27 (25.2)	33 (33.3)
PD90	9 (8.4)	11 (9.7)

The results are presented as the median (interquartile range) or number (%).

MCI = mild cognitive impairment, POCD = postoperative cognitive dysfunction, TICSm = telephone interview for cognitive status-modified.

* *P*<.05, compared with baseline.

† *P*<.05, compared with the control group.

### 3.3. Evaluation of depressive symptoms

**Figure 3.**
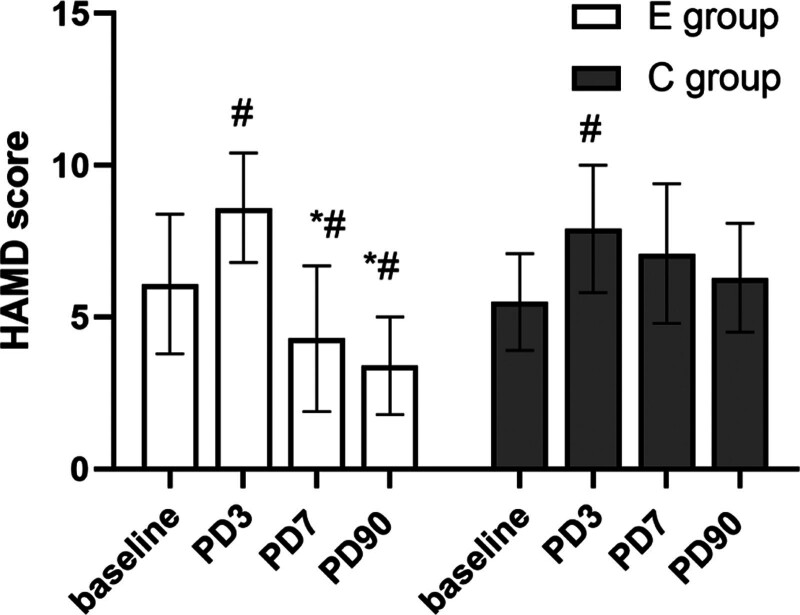
Comparison of postoperative Hamilton Scores between the 2 groups. Compared with the control group, *P < .05; compared with baseline, ^#^*P* < .05. HAMD = Hamilton Depression Scale, PD = postoperative X day.

The incidence of postoperative depressive symptoms at 7- and 90-days post-operation in the E group was lower than in the control group (26.3% vs 47.9, *P* = .026; 13.3 vs 28.4, *P* = .015), and the difference was statistically significant (Table 3). At 3, 7, and 90 days after the operation, Group C had significantly higher HAMD scores than Group E patients. The differences were statistically significant (*P* < .05) (Fig. 3).

**Table 3:** Comparison of the incidence of postoperative depression between the 2 groups.

	Total (%)	E group n (%)	C group n (%)	*P*-value
baseline	69/209 (33.0)	32/108 (29.6)	37/101 (36.6)	.282
PD3	122/201 (60.7)	55/102 (53.9)	67/99 (67.7)	.046
PD7	78/195 (40.0)	26/99 (26.3)	46/96 (47.9)	.026
PD90	36/171 (21.1)	11/83 (13.3)	25/88 (28.4)	.015

The results are presented as the number (%).

PD = postoperative X day.

### 3.4. Evaluation of postoperative pain

The NRS scores on postoperative day 3, along with the effective pressing times and total pressing times of the postoperative analgesic pump in the E group, were significantly lower than those in the C group (*P* < .05). However, there were no significant differences in NRS scores on postoperative days 1 and 2, nor in the required doses of dezocine between the 2 groups (Table 4).

**Table 4:** Comparison of postoperative exercise NRS score, the total number of postoperative analgesic pump presses, the effective number of postoperative analgesic pump presses, and the additional number of desoxine in the 2 groups.

	E group (N = 108)	Control group (N = 101)	*P*-value
NRS-scores at postoperative day 1, mean (SD), score	2.67 ± 0.97	2.84 ± 0.86	.22
NRS-scores at postoperative day 2, mean (SD), score	2.25 ± 1.00	2.46 ± 0.88	.11
NRS-scores at postoperative day 3, mean (SD), score	1.87 ± 0.94	2.13 ± 0.87	.04
Total press times of analgesic pump, median (IQR), time	3.0 (1.0–4.0)	4.0 (2.0–6.0)	.00
Effective press times of analgesic pump, median (IQR), time	1.5 (1.0–3.0)	3.0 (1.0–4.0)	.01
Dezocine n (%)	20 (18.5)	25 (24.8)	.31

The results are presented as the median (interquartile range), mean (SD), or number (%).

NRS = Numerical Rating Scale.

### 3.5. Post-operative quality of recovery and incidence of adverse reactions

**Figure 4.**
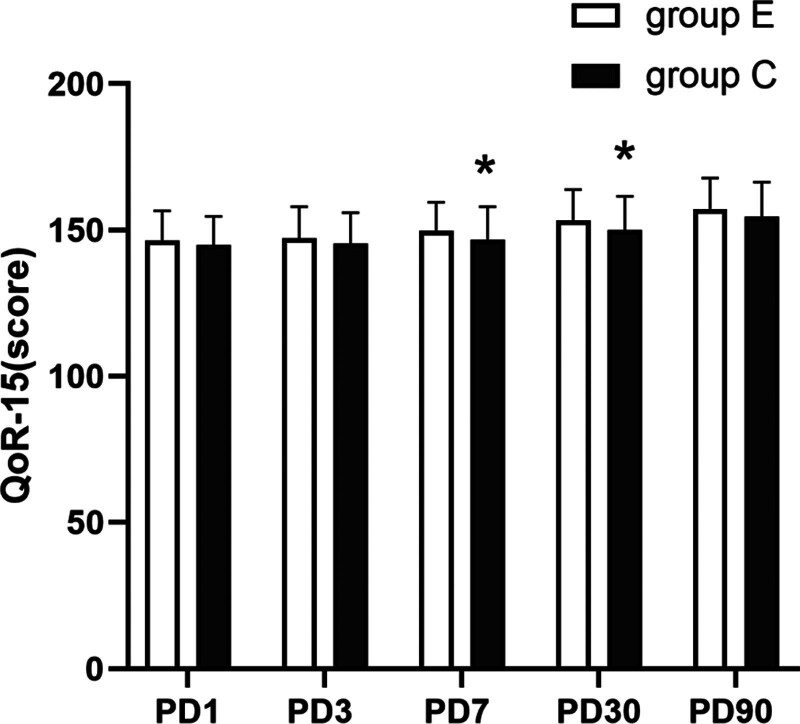
Comparison of QoR-15 between the 2 groups. Compared with the control group, *P < .05. QoR-15 = Quality of Recovery-15 questionnaire, PD = postoperative X day.

The total score of the Quality of Recovery-15 (QoR-15) at both 7 and 30 days after operation was higher in Group E than in Group C, and the difference was statistically significant (*P* < .05) (Fig. 4). Although the incidence of postoperative dizziness and vomiting in Group E was higher than in Group C, the difference was not statistically significant (Table 5).

**Table 5:** The type and incidence of postoperative in-hospitalization adverse events.

Events	E group	C group	*P*-value
Postoperative nausea	16 (14.8)	16 (15.8)	.553
Postoperative vomiting	14 (12.9)	12 (11.9)	.534
Dizziness	16 (14.8)	10 (9.9)	.282
Cystitis	1 (0.9)	0 (0)	.332
Nightmare	3 (2.8)	1 (1.0)	.346
Hallucination	0 (0)	0 (0)	–

Between-group comparison: *P*-value reported based on chi-square test. Data are shown as number (percent).

## 4. Discussion

This study showed that small doses of intraoperative and postoperative esketamine had no significant effect on postoperative delirium or cognitive dysfunction. However, esketamine improved the recovery of executive function, ameliorated the symptoms of postoperative depression, and accelerated patient recovery.

Esketamine, a psychoactive substance with hallucinogenic properties, plays a key role in the onset and development of PND. However, clinical and basic studies have suggested that small doses of esketamine may exert neuroprotective effects. Previous studies have shown that 4 mg/kg of esketamine can effectively reduce cortical oxidative stress, the number of damaged neurons and TUNEL-positive cells in a brain injury model. In contrast, esketamine increased Beclin 1 and LC3 II levels and the number of LC3-positive cells in the affected cortex. Autophagy mediated by AMPK/mTOR-dependent nuclear translocation of TFEB and TFEB/Nrf2-induced antioxidant response were involved in such a phenomenon.^[13]^ In addition, esketamine attenuated isoproterenol-induced cognitive dysfunction and brain damage in rats by activating the mBDNF/TrkB/PI3K signaling pathway.^[29]^

We did not observe the positive effects of esketamine on postoperative delirium and postoperative cognitive dysfunction, which is consistent with the results of an international multicenter randomized controlled trial. The trial showed that a single subanaesthetic dose of ketamine cannot reduce delirium among adults after major surgeries.^[30]^ A phase IV multicenter trial also showed that ketamine is not more effective than placebo in preventing postoperative brain dysfunction and delirium.^[31]^ However, small doses of esketamine effectively reduced the incidence of postoperative delirium and postoperative cognitive dysfunction in several small-sample trials.^[32–34]^ The results of these trials need to be interpreted with caution.

Although small doses of esketamine did not reduce the incidence of postoperative cognitive dysfunction at 7 and 90 days, it was beneficial for the recovery of social executive functioning. Executive functioning is a higher functioning cognitive process that involves the flexible integration of various cognitive processes to achieve specific goals and coherent functioning. The frontal lobe, anterior cingulate gyrus, subcortical structures, basal ganglia, and thalamus are mainly involved in executive functioning.^[35]^ Executive functioning plays a pivotal role in cognition, behavior, and daily activities. Studies have indicated that factors like social engagement marked by tension or conflict can negatively affect executive functioning.^[36]^ Furthermore, researchers have investigated the simultaneous changes in white matter hyperintensities and cognition in older individuals, specifically focusing on episodic memory and executive function.^[37]^ The correlation between executive function and cognition has been examined in different settings, including autism spectrum disorders, revealing significant executive dysfunction in adults with autism that impairs their daily functioning. Moreover, previous studies have unraveled the role of aging in the acute effect of exercise in enhancing cognitive performance, particularly executive function.^[38]^ Understanding the link between exercise-induced changes in executive cognitive function and cerebral perfusion is crucial for developing strategies to promote cognitive health in older adults. Additionally, targeted cognitive interventions, like cognition-specific computerized training, have been investigated as potential remedies for older adults with mild cognitive impairment. These findings underscore the importance of tailored cognitive support for this group.^[39]^ In summary, executive function stands as a critical element of cognition, affected by various factors, including social interactions, neurodegenerative conditions, and aging. In this study, executive functioning was assessed using Part B of Trail Making Test. The Trail Making Test is a test of executive functioning and consists of Part A and Part B. Part B reflects the patient’s ability to maintain attention and switch cognitive goals based on Part A. The shorter the time needed to complete Part B, the better the executive function. Previous studies have indicated that individuals with anxiety and depression may experience impaired social executive functioning. Watkins et al investigated the correlation between rumination and executive functioning in depressed patients, aiming to manage rumination and evaluate its impact on executive functioning. The results revealed that individuals with major depression may exhibit cognitive deficits, particularly in tasks demanding central executive functioning.^[40]^ Nyberg et al measured cognitive functioning, specifically executive functioning, in primary care patients with anxiety disorders, revealing cognitive performance deficits in this group. These findings align with the conclusion of this study that low-dose esketamine can alleviate postoperative depression and accelerate the recovery of social executive functioning.^[41]^ However, further studies are necessary to determine if low-dose esketamine can enhance social executive functioning by alleviating postoperative depression.

The present study showed that one-third of patients with malignancy had a depressive symptom, and approximately half of them developed a postoperative depressive symptom after surgery. Low-dose esketamine improved the postoperative depressive symptoms of elderly patients on days 3, 7, and 90 without increasing psychiatric complications, such as postoperative hallucinations and delirium. This is consistent with the results of previous studies.^[42]^ Multiple studies have indicated that the prevalence of anxiety and depression in cancer patients ranges from 20% to 60%, which is significantly higher compared to the general population. Esketamine not only provides pain relief but also exhibits anxiolytic and antidepressant properties.^[43]^ Rapid improvement in depressive symptoms is observed following intravenous administration, with effects starting within a few hours, peaking at 24 hours, and lasting up to a week.^[44]^ The antidepressant effects of esketamine may be attributed to mechanisms such as increased brain-derived neurotrophic factor (BDNF), activation of the mammalian target of rapamycin complex (mTORC), and reduced inflammation.^[45]^ The observed decrease in postoperative depression scores and incidence of depressive symptoms in Group E may be primarily linked to the use of esketamine. This study focused on elderly patients undergoing major surgery for malignancy, experiencing more severe perioperative stress compared to those undergoing elective surgery, which posed challenges in alleviating depressive symptoms. Despite administering low-dose esketamine, the incidence of depression was as high as 26.3% on postoperative day 7.

Esketamine has demonstrated postoperative antidepressant effects in patients with malignant tumors and has shown potential for treating postoperative depression in various populations of patients. Wang et al found that esketamine does not affect pain control and postpartum depression in pregnant women undergoing cesarean section.^[46]^ Subsequently, a randomized controlled trial by Wang et al demonstrated that esketamine combined with sufentanil reduced the incidence of postpartum depression after cesarean section.^[47]^ Moreover, Min et al reported that postoperative analgesia with esketamine reduced pain, anxiety, and depression in elderly patients undergoing hip arthroplasty.^[48]^ A meta-analysis by Yao et al compared sufentanil alone with the combination of esketamine and sufentanil for patient-controlled intravenous analgesia, indicating potential benefits.^[49]^ Lou et al conducted a meta-analysis of randomized controlled trials examining the effects of esketamine versus placebo on perioperative depression.^[50]^ Niu et al assessed the effects of prophylactic esketamine on postoperative depression and quality of life.^[51]^ Their findings indicated that esketamine can help reduce postoperative depression in different groups of patients undergoing different surgical procedures.

This study introduced several key innovations. To the best of our knowledge, this is the first study to examine the effects of intraoperative and postoperative esketamine on postoperative cognitive function and depression in patients with malignant tumors undergoing radical surgery, a group with a high prevalence of pre- and postoperative depression. We used both intraoperative and postoperative esketamine to measure the effect of repeated doses for a comprehensive evaluation. Previous studies have typically examined only a single intraoperative or postoperative dose of esketamine in combination with other analgesics for patient-controlled intravenous analgesia. Second, the postoperative follow-up was relatively long (90 days) to assess postoperative cognitive function and depression. Previous studies usually had a 1-week follow-up for cognitive function and a 30-day follow-up for depression. Finally, this study used 2 methods to measure cognitive function, including telephone interview for cognitive status-modified (TICSm) and neuropsychological testing. This approach was effective in reducing the possible learning effects of repeated cognitive assessments. Our study had several limitations. First, cognitive function may vary over time, with acute and long-term effects of esketamine showing different effects. Ketamine may cause anticognitive effects during and immediately after intravenous infusion, but cognitive-promoting effects may appear several days or weeks after infusion.^[52]^ Therefore, perioperative low-dose esketamine may have varying effects on postoperative delirium and cognitive dysfunction. This may explain why a low intravenous dose of esketamine does not affect postoperative delirium but enhances partial cognitive function after surgery. However, the results are limited to patients without mental disorders, as we included patients with existing depression and excluded those needing antidepressant medication. Second, depressive symptoms were assessed using the Hamilton Depression Rating Scale 17, which may be prone to recall bias.

## 5. Conclusion

In conclusion, intraoperative low-dose esketamine and postoperative low-dose esketamine combined with sufentanil for PCIA has been shown to improve postoperative analgesia, alleviate postoperative depressive symptoms, and aid in the recovery of social executive ability. However, this approach did not reduce the incidence of postoperative delirium or postoperative cognitive dysfunction.

## Author contributions

**Conceptualization:** Cuifang Huang, Xianlong Xie, Linghui Pan.

**Data curation:** Cuifang Huang, Ruimin Yang.

**Funding acquisition:** Linghui Pan.

**Investigation:** Cuifang Huang, Ruimin Yang, Linghui Pan.

**Methodology:** Cuifang Huang.

**Project administration:** Linghui Pan.

**Resources:** Huijun Dai, Linghui Pan.

**Software:** Cuifang Huang, Linghui Pan.

**Supervision:** Linghui Pan.

**Validation:** Cuifang Huang, Ruimin Yang.

**Visualization:** Cuifang Huang, Xianlong Xie.

**Writing – original draft:** Cuifang Huang.

**Writing – review & editing:** Cuifang Huang, Huijun Dai, Linghui Pan.

## References

[1] Evered L Silbert B Knopman DS. Recommendations for the nomenclature of cognitive change associated with anaesthesia and surgery-2018. Br J Anaesth. 2018;121:1005–12. 30336844 10.1016/j.bja.2017.11.087 PMC7069032

[2] Kim J Shim JK Song JW Kim E-K Kwak YL. Postoperative cognitive dysfunction and the change of regional cerebral oxygen saturation in elderly patients undergoing spinal surgery. Anesth Analg. 2016;123:436–44. 27285000 10.1213/ANE.0000000000001352

[3] Disner SG Beevers CG Haigh EA Beck AT. Neural mechanisms of the cognitive model of depression. Nat Rev Neurosci. 2011;12:467–77. 21731066 10.1038/nrn3027

[4] Hartung TJ Brähler E Faller H. The risk of being depressed is significantly higher in cancer patients than in the general population: Prevalence and severity of depressive symptoms across major cancer types. Eur J Cancer. 2017;72:46–53. 28024266 10.1016/j.ejca.2016.11.017

[5] Sánchez-Rodríguez E Aragonès E Jensen MP. the role of pain-related cognitions in the relationship between pain severity, depression, and pain interference in a sample of primary care patients with both chronic pain and depression. Pain Med. 2020;21:2200–11. 32100028 10.1093/pm/pnz363

[6] Nakata A Irie M akahashi M. Psychological distress, depressive symptoms, and cellular immunity among healthy individuals: a 1-year prospective study. Int J Psychophysiol. 2011;81:191–7. 21740930 10.1016/j.ijpsycho.2011.06.009

[7] Bollettini I Melloni EM Aggio V. Clock genes associate with white matter integrity in depressed bipolar patients. Chronobiol Int. 2017;34:212–24. 27996307 10.1080/07420528.2016.1260026

[8] Zhang M Wang S Wang Z. Associations of affective and cognitive empathy with depressive symptoms among a sample of Chinese college freshmen. J Affect Disord. 2021;292:652–9. 34153836 10.1016/j.jad.2021.05.111

[9] Zhou L Ma X Wang W. Relationship between Cognitive Performance and Depressive Symptoms in Chinese Older Adults: the China Health and Retirement Longitudinal Study (CHARLS). J Affect Disord. 2021;281:454–8. 33360747 10.1016/j.jad.2020.12.059

[10] Voros V Fekete S Tenyi T Rihmer Z Szili I Osvath P. Untreated depressive symptoms significantly worsen quality of life in old age and may lead to the misdiagnosis of dementia: a cross-sectional study. Ann Gen Psychiatry. 2020;19:52. 32944058 10.1186/s12991-020-00302-6 PMC7493324

[11] Trimmel H Helbok R Staudinger T. S(+)-ketamine: Current trends in emergency and intensive care medicine. Wien Klin Wochenschr. 2018;130:356–66. 29322377 10.1007/s00508-017-1299-3 PMC6061669

[12] Hashimoto K. Are “mystical experiences” essential for antidepressant actions of ketamine and the classic psychedelics. Eur Arch Psychiatry Clin Neurosci. 2024. 10.1007/s00406-024-01770-7 PMC12270972 38411629

[13] Tang Y Liu Y Lu H. Esketamine is neuroprotective against traumatic brain injury through its modulation of autophagy and oxidative stress via AMPK/mTOR-dependent TFEB nuclear translocation. Exp Neurol. 2023;366:114436. 37187276 10.1016/j.expneurol.2023.114436

[14] Wang CM Zhang Y Yang YS Lin S He H-F. Effect of esketamine pretreatment on acute sepsis-associated encephalopathy. Exp Neurol. 2024;372:114646. 38070725 10.1016/j.expneurol.2023.114646

[15] Jiang Y Du Z Shen Y Zhou Q Zhu H. The correlation of Esketamine with specific adverse events: a deep dive into the FAERS database. Eur Arch Psychiatry Clin Neurosci. 2023. 10.1007/s00406-023-01732-5 38103077

[16] Jones RR Freeman MP Kornstein SG. Efficacy and safety of esketamine nasal spray by sex in patients with treatment-resistant depression: findings from short-term randomized, controlled trials. Arch Womens Ment Health. 2022;25:313–26. 34973081 10.1007/s00737-021-01185-6 PMC8921149

[17] Ochs-Ross R Wajs E Daly EJ. Comparison of long-term efficacy and safety of esketamine nasal spray plus oral antidepressant in younger versus older patients with treatment-resistant depression: post-hoc analysis of SUSTAIN-2, a Long-Term Open-Label Phase 3 safety and efficacy study. Am J Geriatr Psychiatry. 2022;30:541–56. 34750057 10.1016/j.jagp.2021.09.014

[18] Shen J Song C Lu X. The effect of low-dose esketamine on pain and post-partum depression after cesarean section: A prospective, randomized, double-blind clinical trial. Front Psychiatry. 2022;13:1038379. 36683972 10.3389/fpsyt.2022.1038379 PMC9845877

[19] Ren YL Yuan JJ Xing F Zhu L-N Zhang W. Effects of different doses of esketamine on pain sensitivity of patients undergoing thyroidectomy: a randomized controlled trial. Pain Ther. 2023;12:739–50. 36933139 10.1007/s40122-023-00488-z PMC10199971

[20] Wang P Song M Wang X Zhang Y Wu Y. Effect of esketamine on opioid consumption and postoperative pain in thyroidectomy: a randomized controlled trial. Br J Clin Pharmacol. 2023;89:2542–51. 36967651 10.1111/bcp.15726

[21] Wang M Xiong HP Sheng K Sun X-B Zhao X-Q Liu Q-R. Perioperative administration of pregabalin and esketamine to prevent chronic pain after breast cancer surgery: a randomized controlled trial. Drug Des Devel Ther. 2023;17:1699–706. 10.2147/DDDT.S413273 PMC10259464 37313456

[22] Qiu D Wang XM Yang JJ. Effect of intraoperative esketamine infusion on postoperative sleep disturbance after gynecological laparoscopy: a randomized clinical trial. JAMA Netw Open. 2022;5:e2244514. 36454569 10.1001/jamanetworkopen.2022.44514 PMC9716381

[23] Inouye SK van Dyck CH Alessi CA Balkin S Siegal AP Horwitz RI. Clarifying confusion: the confusion assessment method. A new method for detection of delirium. Ann Intern Med. 1990;113:941–8. 2240918 10.7326/0003-4819-113-12-941

[24] Meybohm P Renner J Broch O. Postoperative neurocognitive dysfunction in patients undergoing cardiac surgery after remote ischemic preconditioning: a double-blind randomized controlled pilot study. PLoS One. 2013;8:e64743. 23741380 10.1371/journal.pone.0064743 PMC3669352

[25] Younan D Wang X Millstein J. Air quality improvement and cognitive decline in community-dwelling older women in the United States: a longitudinal cohort study. PLoS Med. 2022;19:e1003893. 35113870 10.1371/journal.pmed.1003893 PMC8812844

[26] Crooks VC Petitti DB Robins SB Buckwalter JG. Cognitive domains associated with performance on the telephone interview for cognitive status-modified. Am J Alzheimers Dis Other Demen. 2006;21:45–53. 16526589 10.1177/153331750602100104 PMC10833284

[27] He JR Zhang Y Lu WJ. Age-related frontal periventricular white matter hyperintensities and miR-92a-3p are associated with early-onset post-stroke depression. Front Aging Neurosci. 2017;9:328. 29051732 10.3389/fnagi.2017.00328 PMC5633610

[28] Monk TG Weldon BC Garvan CW. Predictors of cognitive dysfunction after major noncardiac surgery. Anesthesiology. 2008;108:18–30. 18156878 10.1097/01.anes.0000296071.19434.1e

[29] Xu G Wang Y Chen Z Zhang Y Zhang X Zhang G. Esketamine improves propofol-induced brain injury and cognitive impairment in rats. Transl Neurosci. 2022;13:430–9. 36561289 10.1515/tnsci-2022-0251 PMC9730546

[30] Avidan MS Maybrier HR Abdallah AB. Intraoperative ketamine for prevention of postoperative delirium or pain after major surgery in older adults: an international, multicentre, double-blind, randomised clinical trial. Lancet. 2017;390:267–75. 28576285 10.1016/S0140-6736(17)31467-8 PMC5644286

[31] Hollinger A Rüst CA Riegger H. Ketamine vs. haloperidol for prevention of cognitive dysfunction and postoperative delirium: a phase IV multicentre randomised placebo-controlled double-blind clinical trial. J Clin Anesth. 2021;68:110099. 33120302 10.1016/j.jclinane.2020.110099

[32] Xiong X Shao Y Chen D Chen B Lan X Shi J. Effect of Esketamine on postoperative delirium in patients undergoing cardiac valve replacement with cardiopulmonary bypass: a randomized controlled trial. Anesth Analg. 2024;139:743–53. 38446699 10.1213/ANE.0000000000006925

[33] Ma J Wang F Wang J. The effect of low-dose Esketamine on postoperative neurocognitive dysfunction in elderly patients undergoing general anesthesia for gastrointestinal tumors: a randomized controlled trial. Drug Des Devel Ther. 2023;17:1945–57. 10.2147/DDDT.S406568 PMC10318106 37408867

[34] Han C Ji H Guo Y. Effect of Subanesthetic Dose of Esketamine on perioperative neurocognitive disorders in elderly undergoing gastrointestinal surgery: a randomized controlled trial. Drug Des Devel Ther. 2023;17:863–73. 10.2147/DDDT.S401161 PMC10039635 36974331

[35] Makuuchi M Kaminaga T Sugishita M. Both parietal lobes are involved in drawing: a functional MRI study and implications for constructional apraxia. Brain research. Brain Res Cogn Brain Res. 2003;16:338–47. 12706214 10.1016/s0926-6410(02)00302-6

[36] Seeman TE Miller-Martinez DM Stein Merkin S Lachman ME Tun PA Karlamangla AS. Histories of social engagement and adult cognition: midlife in the U.S. study. J Gerontol B Psychol Sci Soc Sci. 2011;66:i141–52. 21196438 10.1093/geronb/gbq091 PMC3132769

[37] Maillard P Carmichael O Fletcher E Reed B Mungas D DeCarli C. Coevolution of white matter hyperintensities and cognition in the elderly. Neurology 2012;79:442–8. 22815562 10.1212/WNL.0b013e3182617136 PMC3405254

[38] Lucas S Ainslie P Murrell C. Effect of age on exercise-induced alterations in cognitive executive function: relationship to cerebral perfusion. Exp Gerontol. 2012;47:541–51. 22230488 10.1016/j.exger.2011.12.002

[39] Hill NT Mowszowski L Naismith SL. Computerized cognitive training in older adults with mild cognitive impairment or dementia: a systematic review and meta-analysis. Am J Psychiatry. 2017;174:329–40. 27838936 10.1176/appi.ajp.2016.16030360

[40] Watkins E Brown RG. Rumination and executive function in depression: an experimental study. J Neurol Neurosurg Psychiatry. 2002;72:400–2. 11861707 10.1136/jnnp.72.3.400 PMC1737771

[41] Nyberg J Henriksson M Wall A. Anxiety severity and cognitive function in primary care patients with anxiety disorder: a cross-sectional study. BMC Psychiatry. 2021;21:617. 34886841 10.1186/s12888-021-03618-z PMC8662874

[42] Gan SL Long YQ Wang QY. Effect of esketamine on postoperative depressive symptoms in patients undergoing thoracoscopic lung cancer surgery: a randomized controlled trial. Front Psychiatry. 2023;14:1128406. 37009103 10.3389/fpsyt.2023.1128406 PMC10050377

[43] Bozymski KM Crouse EL Titus-Lay EN Ott CA Nofziger JL Kirkwood CK. Esketamine: a novel option for treatment-resistant depression. Ann Pharmacother. 2020;54:567–76. 31795735 10.1177/1060028019892644

[44] Zarate CA Singh JB Carlson PJ. A randomized trial of an N-methyl-D-aspartate antagonist in treatment-resistant major depression. Arch Gen Psychiatry. 2006;63:856–64. 16894061 10.1001/archpsyc.63.8.856

[45] Halaris A Cook J. The glutamatergic system in treatment-resistant depression and comparative effectiveness of Ketamine and Esketamine: role of inflammation? Adv Exp Med Biol. 2023;1411:487–512. 36949323 10.1007/978-981-19-7376-5_21

[46] Wang Y Zhang Q Dai X. Effect of low-dose esketamine on pain control and postpartum depression after cesarean section: a retrospective cohort study. Ann Palliat Med. 2022;11:45–57. 35144397 10.21037/apm-21-3343

[47] Wang W Xu H Ling B Chen Q Lv J Yu W. Effects of esketamine on analgesia and postpartum depression after cesarean section: a randomized, double-blinded controlled trial. Medicine (Baltim). 2022;101:e32010. 10.1097/MD.0000000000032010 PMC9704928 36451452

[48] Min M Du C Chen X Xin W. Effect of subanesthetic dose of esketamine on postoperative rehabilitation in elderly patients undergoing hip arthroplasty. J Orthop Surg Res. 2023;18:268. 37009879 10.1186/s13018-023-03728-2 PMC10069053

[49] Yao M Fang B Yang J Chen P Chen F. Esketamine combined with sufentanil versus sufentanil in patient-controlled intravenous analgesia: a meta-analysis. Front Pharmacol. 2024;15:1247646. 38384296 10.3389/fphar.2024.1247646 PMC10879558

[50] Lou XJ Qiu D Ren ZY Hashimoto K Zhang G-F Yang J-J. Efficacy and safety of esketamine for perioperative depression in patients undergoing elective surgery: a meta-analysis of randomized controlled trials. Asian J Psychiatr. 2024;95:103997. 38492442 10.1016/j.ajp.2024.103997

[51] Niu G Zheng X Deng B Du YS Mei Y. The effects of prophylactic use of esketamine on postoperative depression and quality of life: a meta-analysis. Minerva Anestesiol. 2024;90:321–9. 38498317 10.23736/S0375-9393.24.17703-6

[52] Malhotra AK Pinals DA Adler CM. Ketamine-induced exacerbation of psychotic symptoms and cognitive impairment in neuroleptic-free schizophrenics. Neuropsychopharmacology. 1997;17:141–50. 9272481 10.1016/S0893-133X(97)00036-5

